# Practicing with what you left

**DOI:** 10.1017/S1478951526103113

**Published:** 2026-06-26

**Authors:** Zongyu Li

**Affiliations:** School of Social Development, East China Normal University, Shanghai, China

I pick up your glasses from the nightstand,
fold the arms, the way you would.
Put them in the drawer. Take them out.
Put them back in.
Day one, I did this seven times.
I fold your grey sweater,
my fingers resting on the worn elbow.
There’s still your shape there,
but not your body’s warmth.
I press my face into the wool –
camphor, tobacco, something I cannot name,
fading.
I remember you saying,
“This one’s got a few more years in it.”
You were wrong about that.
I take the pill bottle,
brown glass, white cap –
your name on the label,
and my handwriting: Take three times a day, with meals.
I pour the tablets into my palm:
little moons, stubborn and light.
Seven. Only seven left.
They couldn’t keep you here,
but I put them back in anyway,
screw the cap tight,
as if something still needs preserving.
Today, it’s the glasses.
Tomorrow, maybe the sweater.
But I’m not ready yet
to touch the pill bottle.
It still sits on the nightstand,
label facing out,
waiting for some hand.

